# Pathogenicity, phylogenomic, and comparative genomic study of *Pseudomonas syringae* sensu lato affecting sweet cherry in California

**DOI:** 10.1128/spectrum.01324-24

**Published:** 2024-09-03

**Authors:** Tawanda E. Maguvu, Rosa J. Frias, Alejandro I. Hernandez-Rosas, Erin Shipley, Greta Dardani, Mohamed T. Nouri, Mohammad A. Yaghmour, Florent P. Trouillas

**Affiliations:** 1 Department of Plant Pathology, University of California, Davis, California, USA; 2 Department of Plant Pathology, Kearney Agricultural Research and Extension Center, Parlier, California, USA; 3 Department of Agricultural, Forest and Food Science, University of Torino, Torino, Italy; 4 Department of Plant Pathology, University of California Cooperative Extension, San Joaquin County, Stockton, California, USA; 5 University of California Cooperative Extension, Kern County, Bakersfield, California, USA; USDA-ARS San Joaquin Valley Agricultural Sciences Center, Parlier, California, USA

**Keywords:** *Pseudomonas syringae*, *Prunus avium*, sweet cherry, pathogenicity, antibiotic resistance, genomics, phylogenetic analysis, bacterial canker, bacterial blast, blossom blast, comparative genomics, genome mining

## Abstract

To gain insights into the diversity of *Pseudomonas syringae* sensu lato affecting sweet cherry in California, we sequenced and analyzed the phylogenomic and genomic architecture of 86 fluorescent pseudomonads isolated from symptomatic and asymptomatic cherry tissues. Fifty-eight isolates were phylogenetically placed within the *P. syringae* species complex and taxonomically classified into five genomospecies: *P. syringae* pv. *syringae*, *P. syringae*, *Pseudomonas cerasi*, *Pseudomonas viridiflava*, and *A*. We annotated components of the type III secretion system and phytotoxin-encoding genes and correlated the data with pathogenicity phenotypes. Intact probable regulatory protein HrpR was annotated in the genomic sequences of all isolates of *P. syringae* pv. *syringae*, *P. syringae*, *P. cerasi*, and *A*. Isolates of *P. viridiflava* had atypical probable regulatory protein HrpR. Syringomycin and syringopeptin-encoding genes were annotated in isolates of all genomospecies except for *A* and *P. viridiflava*. All isolates of *P. syringae* pv. *syringae* caused cankers, leaf spots, and fruit lesions in the field. In contrast, all isolates of *P. syringae* and *P. cerasi* and some isolates of *P. viridiflava* caused only cankers. Isolates of genomospecies *A* could not cause any symptoms suggesting phytotoxins are essential for pathogenicity. On detached immature cherry fruit pathogenicity assays, isolates of all five genomospecies produced symptoms (black–dark brown lesions). However, symptoms of isolates of genomospecies *A* were significantly (*P* < 0.01) less severe than those of other genomospecies. We also mined for genes conferring resistance to copper and kasugamycin and correlated these data with *in vitro* antibiotic sensitivity tests.

**IMPORTANCE:**

Comprehensive identification of phytopathogens and an in-depth understanding of their genomic architecture, particularly virulence determinants and antibiotic-resistant genes, are critical for several practical reasons. These include disease diagnosis, improved knowledge of disease epidemiology, pathogen diversity, and determination of the best possible management strategies. In this study, we provide the first report of the presence and pathogenicity of genomospecies *Pseudomonas cerasi* and *Pseudomonas viridiflava* in California sweet cherry. More importantly, we report a relatively high level of resistance to copper among the population of *Pseudomonas syringae* pv. *syringae* (47.5%). This implies copper cannot be effectively used to control bacterial blast and bacterial canker of sweet cherries. On the other hand, no isolates were resistant to kasugamycin, an indication that kasugamycin could be effectively used for the control of bacterial blast and bacterial canker. Our findings are important to improve the management of bacterial blast and bacterial canker of sweet cherries in California.

## INTRODUCTION

**Fig 1:**
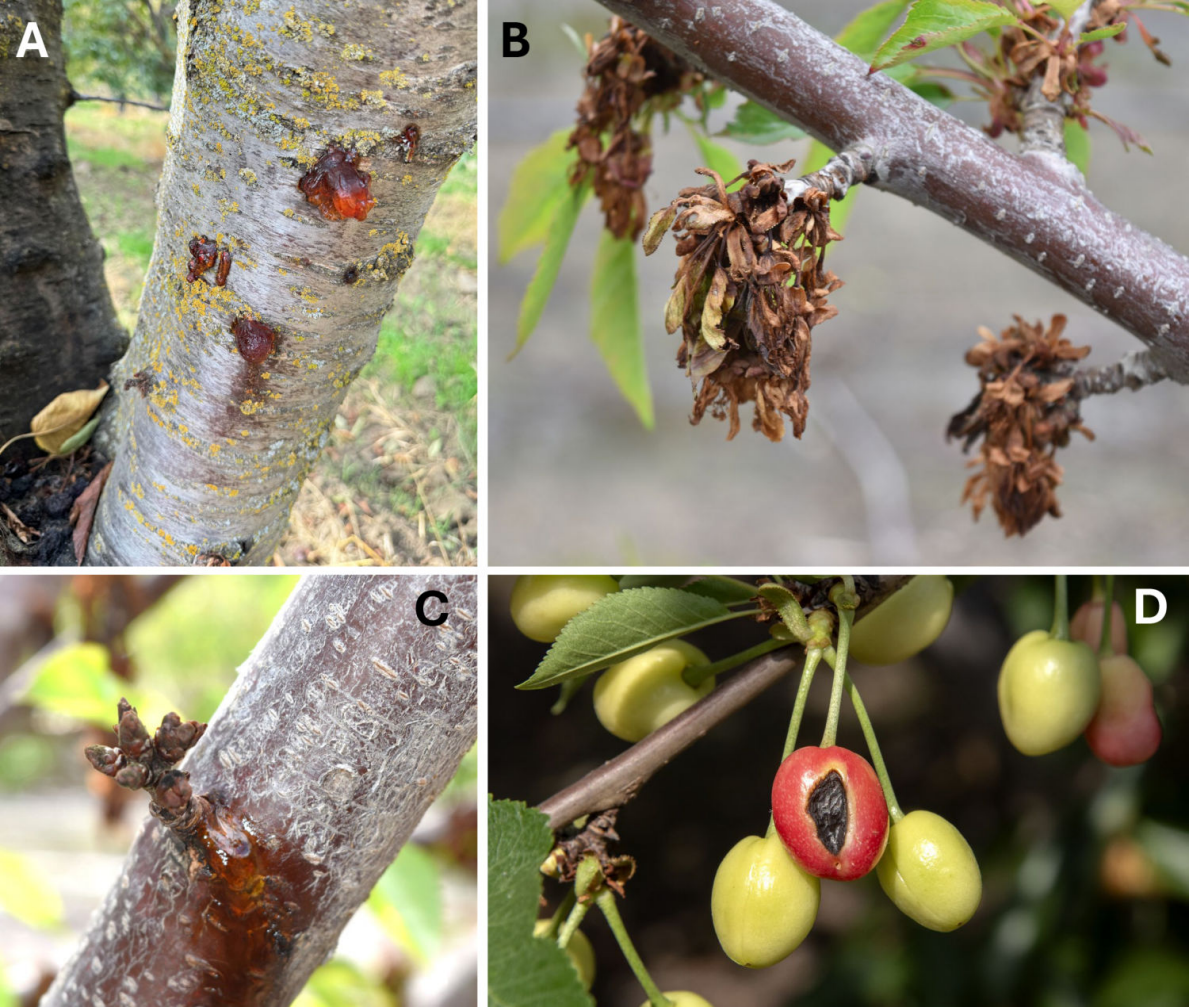
Symptoms of bacterial blast and bacterial canker on sweet cherry. (**A**) Trunk canker with amber-colored gumballs on the margins. (**B**) Blasted flowers; they turn brown and wither while still attached to the plant. (**C**) Dead buds with amber-colored gumming exuding from the base of a dead spur; the bacteria are most likely initiating branch canker. (**D**) Typical fruit rot caused by *Pseudomonas syringae* pv. *syringae* showing a sunken black–dark brown lesion.

Bacterial canker and bacterial blast of stone fruits are two phases of a disease that can severely hamper the production of stone fruits worldwide (1–5). The disease affects many parts of the tree, including flowers, leaves, trunks, and scaffold branches (6). The canker phase of the disease is characterized by small to large irregular cankers with amber-colored gum balls on branches and trunks that can lead to tree death (Fig. 1A). Under the bark, pale, water-soaked, and reddish-brown streaks or flecks extend into the phloem above and below the canker. The blossom blast phase is characterized by flowers that turn dark brown and wither suddenly while still attached to the plant (Fig. 1B). The shoot tip may become necrotic and exude gum; buds infected by the pathogen will mostly die in spring and remain attached to the plant, a condition known as bud death syndrome (Fig. 1C). Developing leaves are also affected by the blast phase; lesions with a chlorotic halo are often observed. Later, the necrotic tissue falls off, producing a shot-hole appearance on the leaves. The impacts of the disease can be devastating, with direct losses resulting from the killing of flowers in the blast phase or from trees declining and dying due to the development of cankers in branches, scaffold limbs, and trunks (7). Bacterial blast has been particularly severe in the past few years (2019–2021 and 2023) in almond and cherry orchards in the Sacramento and San Joaquin valleys of California (5). Developing fruits may also be infected resulting in superficial depressed black–dark brown lesions (Fig. 1D). During the 2024 growing season, there were fewer blast incidences in the Sacramento and San Joaquin valleys of California; however, a widespread occurrence of infected fruits showing black and depressed lesions was observed in orchards with a previous history of blast.

Bacterial blast and canker have been mainly attributed to *Pseudomonas syringae* sensu lato. *P. syringae* is a species complex comprised of several related genomospecies, which are divided into 13 phylogroups based on multi-locus sequence analyses (8, 9). Members of the *P. syringae* species complex exhibit a variety of interactions with plants ranging from benign commensal associations, and opportunistic phytopathogens to host specialized phytopathogens (10, 11). In wild and sweet cherry*, P. syringae* pv. *syringae, Pseudomonas cerasi*, *Pseudomonas amygdali* pv. *morsprunorum* (formerly *P. syringae* pv. *morsprunorum* race 1), and *Pseudomonas avellanae* (formerly *P. syringae* pv. *morsprunorum* race 2) have been reported to be associated with either bacterial canker or bacterial blast (1, 3, 12). In California, currently, there are no reports of *P. amygdali*, *P. avellanae*, and *P. cerasi* isolates associated with bacterial canker/blast of cherry. Only *P. syringae* pv. *syringae* has been reported to be associated with bacterial blast and canker of sweet cherry in California, and the occurrence of other pathogenic *Pseudomonas* species is unknown. The taxonomic classification of the *P. syringae* species complex has been mired by several complexities resulting from low-resolution analytic methods (13). Considering that the most comprehensive analyses of the diversity of *P. syringae* associated with sweet cherries in California were done more than two decades ago (3), it is worth revisiting this issue using high-resolution taxonomic and classification methods like whole genome-based phylogenomic analyses. Recently, using whole genome sequencing-based phylogenomic analyses complemented with pathogenicity assays, we demonstrated the presence and pathogenicity of *P. cerasi* and *Pseudomonas viridiflava* in almond orchards in California (5). These findings suggest more species are possibly associated with other *Prunus* species such as sweet cherry in California. Good knowledge of pathogen diversity and the associated biology is important for the implementation of effective control strategies. The recent advent and affordability of whole genome sequencing allow for whole genome-based taxonomic classification of isolates. Whole genome sequencing-based phylogenomic analyses greatly improve the identification of species, as they elucidate the functional profiles of taxonomic groups and resolve ambiguities in the phylogeny of higher taxa that are difficult using traditional approaches (14–16).

*P. syringae* sensu lato is a well-studied phytopathogen, and what makes *P. syringae* a successful phytopathogen has been well described (17). It has an array of phytotoxins and type III secretion system effectors, which are critical to its pathogenesis (18, 19). Advances in genome sequencing and bioinformatics analyses allow for such virulence factors to be mined from genomic sequences, and putative phytopathogens can be identified based on genomic features (20). This is useful particularly when dealing with a large collection of isolates; however, such genomic predictions can only be robust when complemented with phenotypic pathogenicity tests. Genomic predictions do not always guarantee the predicted phenotypes; deletion of the four effectors predicted to be critical for cherry bacterial canker pathogens (*hopAR1*, *hopBB1*, *hopBF1*, and *hopH1*) did not cause a significant reduction in disease symptoms (21). Hence, genomic predictions must be interpreted together with observed phenotypic data. In this study, we sequenced and comprehensively analyzed the genomic architecture and phylogeny of 86 fluorescent pseudomonads isolated from symptomatic and asymptomatic cherry tissue. Genome mining was complemented with phenotypic pathogenicity studies to comprehensively identify fluorescent pseudomonads pathogenic to sweet cherry in California. A combination of the predicted virulence factors was correlated with the pathogenicity phenotypes to understand the variation of pathogenicity among strains. From the genomic sequences, we also mined for genes conferring resistance to antibiotics, and we correlated these data with the observed *in vitro* antibiotic sensitivity tests.

## MATERIALS AND METHODS

During the winter and spring of 2022–2023, 300 isolates of fluorescent pseudomonads were isolated from symptomatic and asymptomatic flowers, dormant vegetative and flower buds, leaves, twigs, and bark tissues of cherry, from several orchards in the Sacramento and San Joaquin valleys of California. Data S1 provides detailed information on the isolates’ metadata. Isolation was done by plating on King’s B (KB) medium containing 50 µg/mL of cycloheximide (KBC) as previously described (5). Bacterial colonies that exhibited fluorescence on the KBC medium were collected irrespective of colony appearance. From the collection, a total of 86 isolates from both symptomatic and asymptomatic tissues were selected for sequencing. The genomic DNA of the selected isolates was extracted and purified using the FastDNA Spin Kit (MP Biomedicals, Irvine, CA) following the manufacturer’s protocol. Agarose gel electrophoresis and NanoDrop spectrophotometry (ND-100, NanoDrop Technologies Inc, Wilmington, DE, USA) were used to determine the integrity and purity of the resultant DNA, respectively. The DNA was submitted for whole genome sequencing to the Microbial Genome Sequencing Center (MIGS, Pittsburgh, PA), and paired-end reads (2 × 151 bp) were generated from Illumina sequencing.

### Genome assembly and annotations

We received high-quality raw reads (*Q* > 30), and *de novo* assembling of the quality reads was performed using SPAdes version 3.13.0 (22). CheckM and QUAST were used to assess the quality metrics of the assembled genomes (23, 24). Annotations were done using RAST with default settings (25). The analysis was performed on the Kbase platform (26). Based on CheckM results, genomes with <2% contamination and ≥99% completeness were used for downstream analyses. Genomes that did not meet the stated quality metrics were discarded. Resistance Gene Identifier was utilized to predict resistomes in the genomic sequences using homology and Single-nucleotide polymorphism (SNP) models on the Comprehensive Antibiotic Resistance Database with default settings (27), and the Virulence Factors of Pathogenic Bacteria database was used to identify virulence factors (28).

### Whole genome-based phylogenomic analyses

The Type Strain Genome Server was used for pairwise comparison of our assembled genomes and the phylogenetically related type strains using the Genome Blast Distance Phylogeny (29). Inter-genomic distances were inferred using the trimming algorithm and distance formula *d*_5_, with 100 replicates (30). Digital DNA-to-DNA hybridization (dDDH) values and confidence intervals were calculated using the recommended settings of Genome-to-Genome Distance Calculator (GGDC) 2.1. The resulting inter-genomic distances were used to infer a balanced minimum evolutionary tree with branch support via FASTME 2.1.4, including Subtree pruning and regrafting (SPR) post-processing (31). Trees were rooted at the midpoint and visualized with PhyD3 (32). The resulting tree was imported to the interactive tree of life for display and addition of annotation features (33). The genome taxonomy database toolkit (GTDB-Tk) was also used to assign taxonomy based on the genome taxonomy database (34). The GTDB-Tk uses average nucleotide identity (ANI) for taxonomic classification. The analyses included at least one representative genome sequence of the established *P. syringae* phylogroups when a genome sequence could be attained publicly.

### Pathogenicity tests

Based on the phylogenomic analyses, 31 isolates representing the five identified genomospecies within the *P. syringae* species complex were selected for pathogenicity testing. The selection of isolates was based mainly on the intraspecies variation of the type III secretion system (T3SS) and phytotoxin-encoding genes. Sixteen isolates were selected for *P. syringae* pv. *syringae*, one isolate from genomospecies *A*, three isolates from *P. syringae*, four isolates from *P. cerasi*, and seven isolates from *P. viridiflava*. In addition, nine isolates’ representatives of the other fluorescent pseudomonads [*Pseudomonas fluorescence* (2), *Pseudomonas carnis*, *Pseudomonas putida* (2), *Pseudomonas koreensis*, *Pseudomonas proteolytica* (2), and isolate PS1010] were also tested. Phosphate-buffered saline (PBS) was used as a control. Bacterial suspensions of each selected isolate were prepared from 30-h-old KBC colonies in 12-mL Falcon tubes containing sterile PBS. The inoculum concentration was adjusted to approx. 10^8^ CFU/mL (90% transmission at OD600) using a DU730 Life Sciences UV/Vis Spectrophotometer (Beckman Coulter Inc., Fullerton, CA, USA). A similar procedure was used for all other inoculation experiments.

For the canker field experiments at the Kearney Agricultural Research and Extension Centre (KARE), the inoculation sites on 6-year-old *Prunus avium* cv. Bing branches were first disinfected with 90% ethanol in November 2023 and March 2024. A sterile nail was then used to puncture the bark and create three wounds/branch/tree for each isolate. The experiment was laid out in six four-tree blocks with each isolate contained in each block, resulting in a total of six tree replicates per isolate. Bacterial suspensions were sprayed onto the wounds using a mist spray bottle, and PBS was used as a control. To mimic natural infections, the wounds were not wrapped after inoculations. Disease evaluations were done 2 weeks post-inoculation, and monitoring continued for another successive 6 weeks. The pathogenicity of the isolates was determined by scoring for the presence of gum exudates that were produced on the bark at the punctured sites (see Fig. S1A through C). Data are presented for disease severity, which is the average number of gum exudates produced for all six replications. In addition, disease incidence ratings are provided as the % of branches that showed at least one gumming from the inoculation sites. A 100% incidence means that the branches of all six replicate trees have at least one inoculated point gumming. To fulfill Koch’s postulates, active canker margins were plated onto the KB medium, and isolated fluorescent bacteria were subjected to colony PCR as previously described (5).

For leaf pathogenicity tests in the field, leaflets of cv. Bing at KARE were inoculated on 22 March and 3 April 2024. The experiment was laid out in six four-tree blocks with each isolate contained in each block, resulting in a total of six tree replicates per isolate. Leaflets from a single vegetative spur (≈6 leaves) were used per tree replicate. Leaflets were spray-inoculated as described above for cankers, the disease was evaluated 2 weeks post-inoculation, and monitoring continued for another 2 successive weeks. For this, leaflets from each of the six treated blocks (6 leaves/vegetative spur × 6 blocks) were assessed for leaf spots/lesions with a chlorotic halo: typical symptoms of *P. syringae* infection ( Fig. S1D). Data are presented as the average number of leaf spots/lesions for the March and April inoculations.

We also inoculated immature fruits cv. Bing on sweet cherry trees in the field. Twenty immature fruits were tagged per isolate. The inoculum was sprayed using a mist spray bottle to cover the surface of each of the 20 immature cherry fruits. In this experiment, no wounding of the fruit was done. Data on the occurrence of fruit lesions were collected after 7 days, and monitoring continued for another 2 weeks. PBS was used as a control.

**Fig 2:**
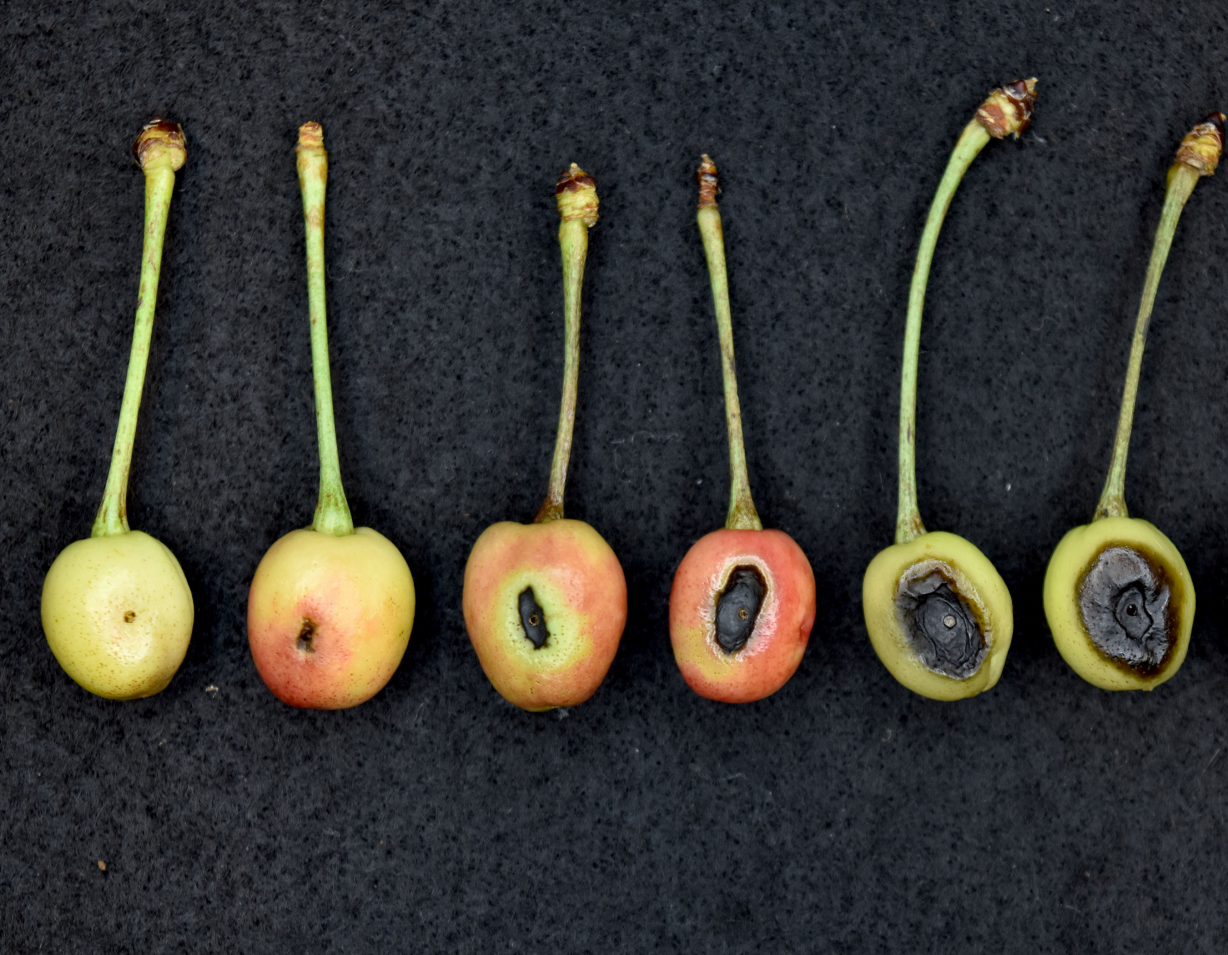
Detached immature fruit pathogenicity test. Rating scale for lesions/rot on the fruits. The image shows severity score ratings (0-5) from left to right.

For laboratory assays on cherry fruits, immature, green cherry fruits were freshly collected and washed thoroughly under running tap water for 5 min and then dipped into 10% commercial bleach for 5 min. The fruits were then rinsed four times with sterile distilled water and air-dried in a sterile flow bench. Sterile toothpicks were used to puncture the immature fruits, and 5 µL of the inoculum was applied to the wounded area using a pipette. For each isolate, 10 fruits were inoculated. PBS was used as a control. The fruits were placed on a raised plastic mesh inside a transparent crisper box containing paper towels wetted with sterile distilled water and incubated at 25°C under natural light conditions. Disease ratings were done by assigning a score of 0–5 to the observed lesions on the inoculated site. Figure 2 shows representative lesions assigned to each score. Data were recorded daily for a period of 7 days. This experiment was done twice. For this experiment, a subset of *P. syringae* pv. *syringae* (five isolates), *P. syringae* (three isolates), genomospecies *A* (one isolate), *P. cerasi* (four isolates), and *P. viridiflava* (six isolates) were used.

### Kasugamycin and copper sensitivity tests

All isolates phylogenetically placed with the *P. syringae* species complex were tested for their sensitivity to kasugamycin and copper, two bactericides used to manage bacterial blast in California. To evaluate the toxicity of kasugamycin (Kasumin 2L, Arysta LifeScience, Cary, NC), the agar dilution plate method was used. Nutrient agar was amended with 100 µg/mL kasugamycin, the labeled rate of the product. Unamended plates were used as a control. For copper (Champ WG, Nufarm Americas Inc), nutrient agar was amended with 100, 200, 300, or 400 µg/mL metallic copper equivalent (MCE). Aliquots of 2-µL bacterial suspensions at 10^8^ CFU were inoculated onto control and bactericide-amended plates. Plates were incubated for 48 h at 25°C and visually inspected for bacterial growth. For kasugamycin, growth on amended plates was considered resistance. For copper, resistance was defined as visible growth at concentrations that inhibited the growth of the other isolates. The experiment was done three times.

### Statistical analyses

For statistical analyses, the Kruskal–Wallis test was used followed by *post hoc* Dunn’s test using the Bonferroni correction method to correct the α value.

## RESULTS AND DISCUSSION

### Genome quality metrics and whole genome sequencing-based phylogenomic analyses

**Fig 3:**
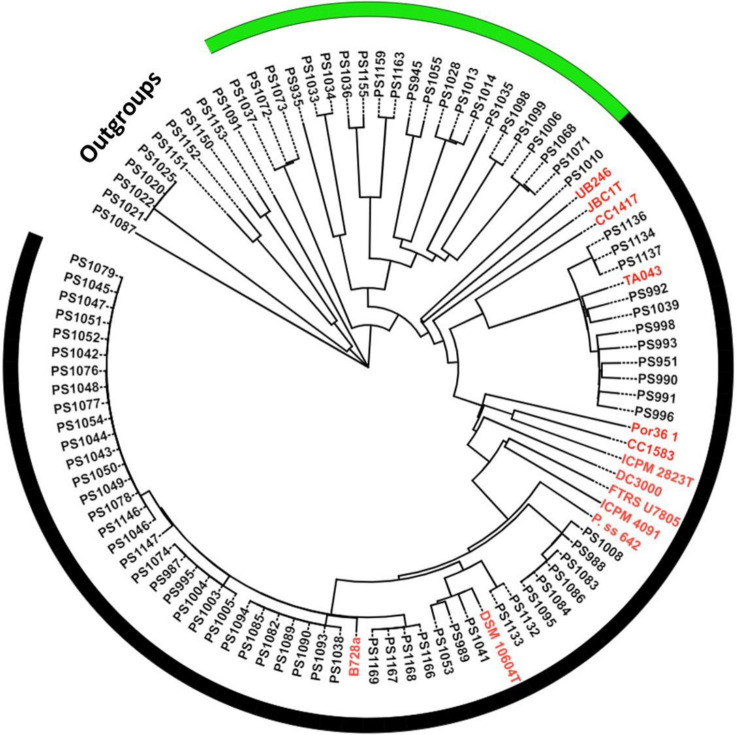
Balanced minimum evolutionary tree inferred from inter-genomic distances of the whole genome sequences calculated using GGDC 2.1. Using phylogenomic analyses, fluorescent pseudomonads isolated from sweet cherry clustered into two main clades designated as *P. syringae* species complex (indicated by the black outer border) and other fluorescent pseudomonads (indicated by the green outer border). The outgroups consisted of non-pseudomonad isolates. Isolates highlighted in red are representatives of the established *P. syringae* phylogroups.

**Fig 4:**
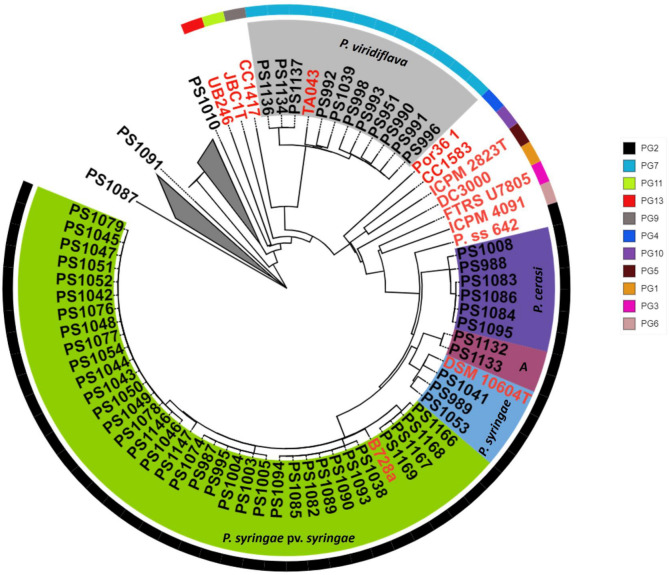
A close-up of the phylogenomic analyses of the *P. syringae* species complex from Fig. 3. Isolates from this study are highlighted in black, and isolates highlighted in red are representatives of the established *P. syringae* phylogroups. Isolates from this study fall into two main phylogroups (PG) PG2 and PG7 (see key). They were taxonomically classified into at least five genomospecies based on dDDH; *P. syringae* pv. *syringae*, *P. syringae*, *A*, *P. cerasi,* and *P. viridiflava*.

We isolated and sequenced 86 fluorescent pseudomonads from symptomatic and asymptomatic cherry tissues. Based on CheckM results, 79 genomes had <2% contamination and ≥99% completeness. Data S2 shows the quality metrics of all the assembled genomes. Genomes with ≥2% contamination or <99% completeness were not used for downstream analyses. Phylogenomic analyses of pseudomonads bacteria from cherry tissues identified a high diversity of *Pseudomonas* species (Data S3 and S4). The balanced minimum evolutionary tree inferred from inter-genomic distances calculated using the Genome-to-Genome Distance Calculator resulted in isolates clustering into two main phylogenomic clades designated *P. syringae* species complex (58 isolates) and other fluorescent pseudomonads (21 isolates) (Fig. 3). The *P. syringae* species complex clade is known to include plant pathogenic bacteria. However, members of this species complex exhibit a variety of interactions with plants ranging from benign commensal associations and opportunistic phytopathogens to host specialized phytopathogens (10, 11). Thus, it is critical to validate the pathogenicity of isolates from this group. Taxonomic classification of the isolates belonging to the *P. syringae* species complex identified at least five genomospecies: *P. syringae* pv. *syringae* (35 isolates), *P. syringae* (3 isolates), *P. cerasi* (6 isolates), *P. viridiflava* (11 isolates), and a genomospecies designated *A* (2 isolates) (Fig. 4). Genomospecies *A* is used solely to distinguish isolates in this study, and it is not related to the *Pseudomonas A* species reported by reference (5). dDDH and ANI were used for the taxonomic classification of the isolates. dDDH and ANI use whole genome sequences for the classification of isolates. Whole genome sequencing-based phylogenomic analyses greatly improve the identification of species, as they elucidate the functional profiles of taxonomic groups and resolve ambiguities in the phylogeny of higher taxa that are difficult to identify using traditional approaches (14–16). *P. syringae* pv. *syringae* and *P. syringae* are two different genomospecies with *P. syringae* clustering with *P. syringae* type strain (*P. syringae* KCTC 12500). Thus, based on phylogenomic analyses, *P. syringae* pv. *syringae* including the model strain B728a are not *P. syringae* genomospecies. Several studies have clarified the taxonomic classification of members of the *P. syringae* species complex and have shown that *P. syringae* pv. *syringae* shares less than 70% dDDH with the *P. syringae* type strain (5, 13). In this study, we will concentrate more on the pathogenicity of isolates rather than clarification of the taxonomic status. However, we name our isolates based on the International Code of Nomenclature of Prokaryotes-established species cutoff of 70% based on dDDH.

### Genome mining for virulence factors and antibiotic resistance determinants

**Fig 5:**
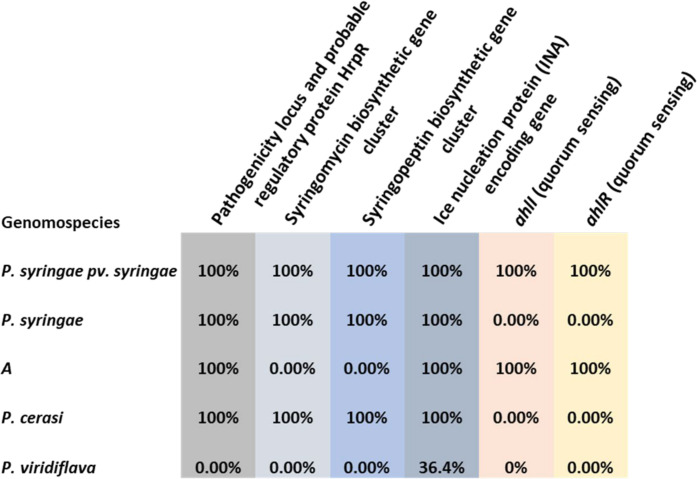
Some of the virulence determinants and epiphytic fitness-related genes annotated from the genomic sequences of isolates belonging to the *P. syringae* species complex. The % represents the % of isolates with the corresponding gene annotated from their genomic sequences from each of the five identified genomospecies (*P. syringae* pv. *syringae*, *P. syringae*, *A*, *P. cerasi,* and *P. viridiflava*).

The T3SS and the phytotoxins are the key virulence factors of *P. syringae* sensu lato (18, 19). These genes can be easily mined from genomic sequences thereby identifying putative phytopathogens based on the genomic content. Genomic mining revealed that all isolates belonging to the *P. syringae* species complex harbored components of the T3SS although there were inter- and intraspecies variations in the components present among isolates (Data S5). An intact pathogenicity locus, probable regulatory protein HrpR was annotated in all isolates of the genomospecies *P. syringae* pv. *syringae*, *P. syringae*, *P. cerasi*, and *A* (Fig. 5). In contrast, no intact pathogenicity locus, probable regulatory protein HrpR was annotated from the genome sequences of genomospecies *P. viridiflava* (Fig. 5). *P. viridiflava* is considered an outsider of the *P. syringae* species complex due to its atypical regulatory pathogenicity locus and reliance on pectate lyase (PL) as the main virulence determinant (35). PL macerates plant tissue resulting in soft rot, and *P. viridiflava* is mainly a soft rot pathogen (36, 37). *P. viridiflava* had more genes encoding for PL and its precursors in contrast to the other genomospecies. *P. syringae* species have been shown to produce several phytotoxins that can be co-ordinately regulated by T3SS to aid pathogenesis, albeit secreted independently from T3SS (38). Toxins like syringomycin and syringopeptin have been shown to have membrane disruption and ion leakage activities (38). Genome mining revealed the presence of genes encoding for syringomycin and syringopeptin in the genomic sequences of all isolates belonging to the genomospecies *P. syringae* pv. *syringae*, *P. syringae*, and *P. cerasi* (Fig. 5). In contrast, isolates of genomospecies *A* and *P. viridiflava* had no genes encoding for syringomycin and syringopeptin annotated from their genomic sequences (Fig. 5). Apart from the T3SS and phytotoxins, ice nucleation protein is considered an important feature for the success of some phytopathogenic *P. syringae* strains. Bacterial ice nucleation is associated with the ability to cause frost damage that may lead to water and nutrient release from plants and could create openings on the plant surface to facilitate bacterial entry (17). In cherries, the freezing/thawing process has been shown to facilitate the passive ingress of *P. syringae* into leaves (39). Genome mining determined the presence of genes encoding for ice nucleation activity (INA) proteins in all isolates of genomospecies *P. syringae* pv. *syringae*, *P. syringae*, *P. cerasi*, and *A* (Fig. 5). In contrast, only 36% of isolates belonging to the genomospecies *P. viridiflava* had genes encoding for INA annotated from their genomic sequences (Fig. 5). The leaf surface is a hostile environment, and aggregated bacterial cells tend to survive better than solitary cells (40). For *P. syringae*, the establishment of large epiphytic populations is a prerequisite for disease occurrence (41). Quorum sensing positively regulates several traits that are crucial for epiphytic fitness, and cells in large aggregates are resistant to environmental stress to which they are exposed during the epiphytic phase (42, 43). As a result, we also mined for quorum-sensing-related genes. *N*-Acyl homoserine lactone (AHL) is the quorum-sensing signal molecule in *P. syringae* pv. *syringae,* and its production requires the expression of the AHL synthase gene, *ahlI*, and the AHL regulator gene, *ahlR* (43, 44). *ahlI* and *ahlR* were annotated in genome sequences of all isolates of the genomospecies *P. syringae* pv. *syringae* and *A* (Fig. 5). No other isolates had these genes.

We also mined for genes conferring resistance to antibiotics with a bias toward genes conferring resistance to copper and kasugamycin, the two most widely used bactericides/antibiotics in California cherry production. No isolates harbored genes conferring resistance to kasugamycin (Table 1 ). Resistance to kasugamycin is conferred by altering the target methylated nucleotides in 16S rRNA and the disruption of the KsgA methyltransferase or inactivation of the molecule (kasugamycin) by *aac(2′)-IIa* encoding 2′-*N*-acetyltransferase (45–47). Manual inspection revealed that there were no alterations in the kasugamycin target methylated nucleotides in 16S rRNA or any evidence of disruption of the KsgA methyltransferase. Moreover, no isolates had the *aac(2′)-IIa* annotated from their genomic sequence (Table 1). Genome mining also detected the presence of CtpV, a putative copper exporter in 45.7% and 35.5% of isolates belonging to the genomospecies *P. syringae* pv. *syringae* and *P. viridiflava*, respectively (Table 1). The CtpV is a putative copper exporter that reduces the impacts of copper toxicity and is involved in the virulence of *Mycobacterium tuberculosis* (48). It has been shown to positively correlate with the copper resistance phenotype of *P. syringae* pv. *syringae* isolates from almonds in California (5). No other genetic determinants of copper resistance were detected from the genomic sequences.

**TABLE 1:** Correlations of kasugamycin and copper resistance genotypes with their phenotypes

		Copper resistance phenotype		
Genomospecies	Annotated copper resistance genotype (*ctpV*)	200 µg/mL MCE	300 µg/mL MCE	400 µg/mL MCE
*P. syringae* pv. *syringae*	16/35 isolates (47.5%)	100%	47.50%	47.50%
*P. syringae*	0/3 isolates (0%)	100%	0%	0%
*A*	0/2 isolates (0%)	100%	0%	0%
*P. cerasi*	0/6 isolates (0%)	100%	0%	0%
*P. viridiflava*	5/11 isolates (45.5%)	100%	45.50%	45.50%

		Kasugamycin resistance phenotype	
Genomospecies	Annotated kasugamycin resistance genotype	75 µg/mL	100 µg/mL
*P. syringae* pv. *syringae*	0/35 isolates (0%)	0%	0%
*P. syringae*	0/3 isolates (0%)	0%	0%
*A*	0/2 isolates (0%)	0%	0%
*P. cerasi*	0/6 isolates (0%)	0%	0%
*P. viridiflava*	0/11 isolates (0%)	0%	0%

### Pathogenicity tests

**Fig 6:**
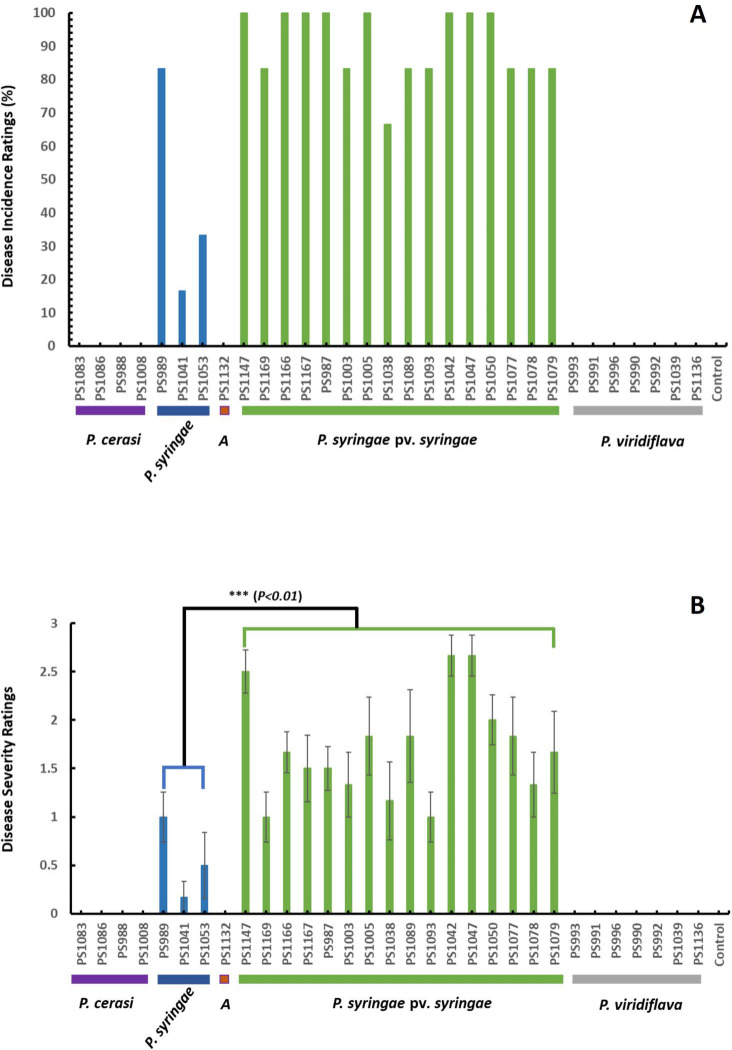
Canker pathogenicity tests in November 2023. (**A**) % of disease incidences; (**B**) disease severity ratings; data are the average of all replications, and error bars indicate ±standard deviation of the mean.

In the canker pathogenicity tests conducted in November 2023, the results indicated that only isolates of the genomospecies *P. syringae* pv. *syringae* and *P. syringae* were pathogenic (Fig. 6A and B). For genomospecies *P. syringae* pv. *syringae*, eight isolates recorded 100% disease incidences, with seven isolates recording 83.3% disease incidences and one isolate recording 66.6% (Fig. 6A). In contrast, the three isolates of genomospecies *P. syringae* recorded disease incidences of 83.3%, 33.3%, and 16.6% (Fig. 6A). Isolates of *P. syringae* pv. *syringae* recorded the highest disease severity ratings with a range of 1 ± 0.26–2.66 ± 0.21 (Fig. 6B). In contrast, isolates of the genomospecies *P. syringae* had severity ratings ranging from 0.17 ± 0.17 to 1 ± 0.26 (Fig. 6B). Isolates of the genomospecies *P. syringae* pv. *syringae* were significantly more aggressive (*P* < 0.01) than isolates of the genomospecies *P. syringae* (Fig. 6B). Moreover, the results demonstrated intraspecies variation in the incidence and severity of the disease caused by the isolates (Fig. 6A and B). The intraspecies variation in the degree of aggressiveness is common among members of the *P. syringae* species complex and can be attributed to slight genetic variations and adaptations to host plants (3–5). Isolates of the other fluorescent pseudomonads that did not cluster with members of the *P. syringae* species complex as well as isolate PS1010 were not pathogenic in this assay and all the other experiments.

**Fig 7:**
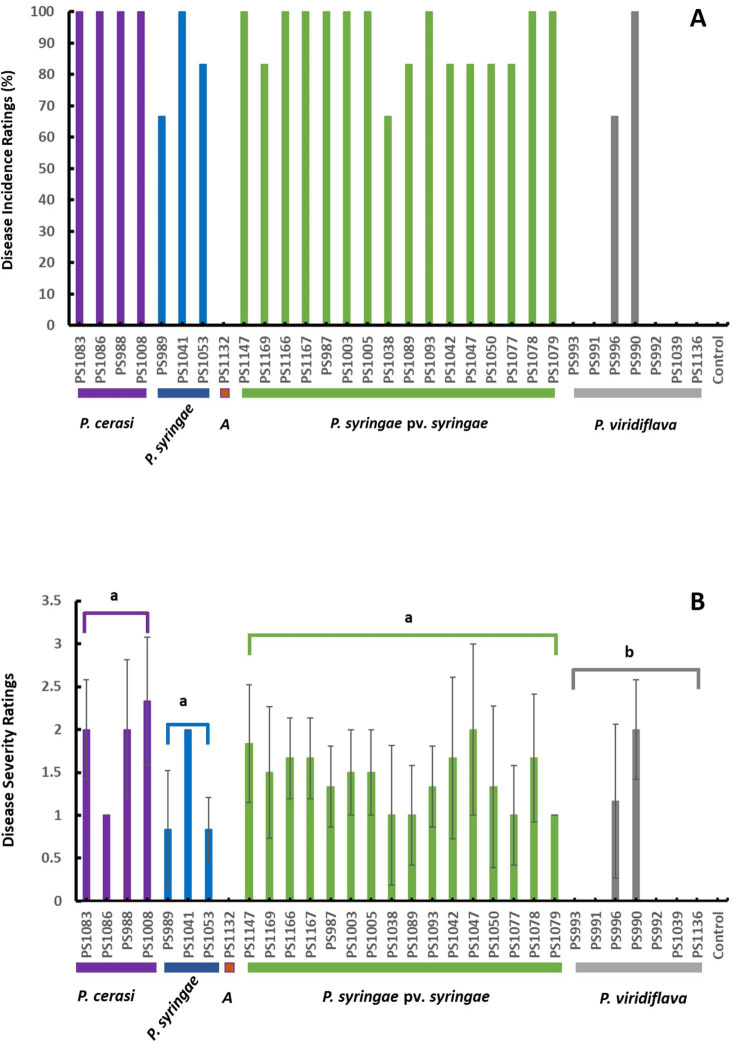
Canker pathogenicity tests in March 2024. (**A**) % of disease incidences; (**B**) disease severity ratings; data are the average of all replications, and error bars indicate ±standard deviation of the mean. Different letters indicate significant differences (*P* < 0.01) in the severity ratings among isolates of the different genomospecies.

In contrast to the experiment conducted in November 2023, the results of the experiment conducted in March 2024 indicated that besides isolates of the genomospecies *P. syringae* pv. syringae and *P. syringae*, all isolates of *P. cerasi* and two isolates of *P. viridiflava* were pathogenic (Fig. 7A and B). All isolates of genomospecies *P. cerasi* recorded 100% disease incidences (Fig. 7A). The three isolates of *P. syringae* recorded 100%, 83.3%, and 66% disease incidences, which were relatively higher than the disease incidences recorded in November 2023 (Fig. 6A and 7A). Nine isolates of the genomospecies *P. syringae* pv. *syringae* recorded 100%, six isolates recorded 83.3%, and a single isolate recorded 66.7% disease incidence (Fig. 7A). Three of the four *P. cerasi* isolates had severity ratings of at least 2 ± 0.8, while the remaining isolate had a severity rating of 1 (Fig. 7B). Two isolates of genomospecies *P. syringae* had a severity score of 0.83, and the third isolate had a severity score of 2 (Fig. 7B). Isolates of the genomospecies *P. syringae* pv. *syringae* had severity rating scores ranging from 1 ± 0.8 to 2 ± 0.9 (Fig. 7B). The two pathogenic *P. viridiflava* isolates had severity ratings of 1.17 ± 0.9 and 2 ± 0.6 (Fig. 7B). Isolates of the genomospecies *P. syringae* pv. *syringae*, *P. cerasi,* and *P. syringae* had severity ratings significantly higher (*P* < 0.01) than those of *P. viridiflava* (Fig. 7B). There were no significant differences (*P* > 0.05) in the severity ratings of the isolates of genomospecies *P. syringae* pv. *syringae*, *P. cerasi*, and *P. syringae*. However, it is important to note that the gumballs produced at the inoculation sites by isolates of the genomospecies *P. syringae* and *P. viridiflava* were relatively smaller than those produced by isolates of the genomospecies *P. syringae* pv. *syringae* and *P. cerasi* (see Fig. S1B). Given that no gumming was observed on control treatments, the observed gummosis was a specific response triggered by biotic aggression. Considering that the gum consists of polyphenols and volatile compounds with antibacterial properties (49), we surmise that the degree of gummosis correlates with the aggression of the phytopathogen. In a canker pathogenicity study in almonds, *P. cerasi* isolates showed a similar trend where they were not pathogenic in winter experiments, but they were pathogenic in experiments conducted at the end of winter/beginning of spring (5). The possible rationale could be their virulence factors *in planta* are triggered at warmer temperatures. Alternatively, changes in plant metabolites at the beginning of spring might trigger the expression of their virulence factors. Transcriptomics and metabolomics studies might shed more light on how this host–microbe interaction results in disease development during particular time points (winter vs spring). There was no clear combination of T3SS, which was necessary for symptom development. However, the absence of phytotoxin-encoding genes in isolates of genomospecies *A* might explain their inability to cause any symptoms. Genomospecies *A* had components of T3SS similar to genomospecies *P. syringae* but could not cause any symptoms. The lack of clear combinations of T3SS necessary for symptom development was not surprising as effectors have been described as collectively essential but individually dispensable (50).

**Fig 8:**
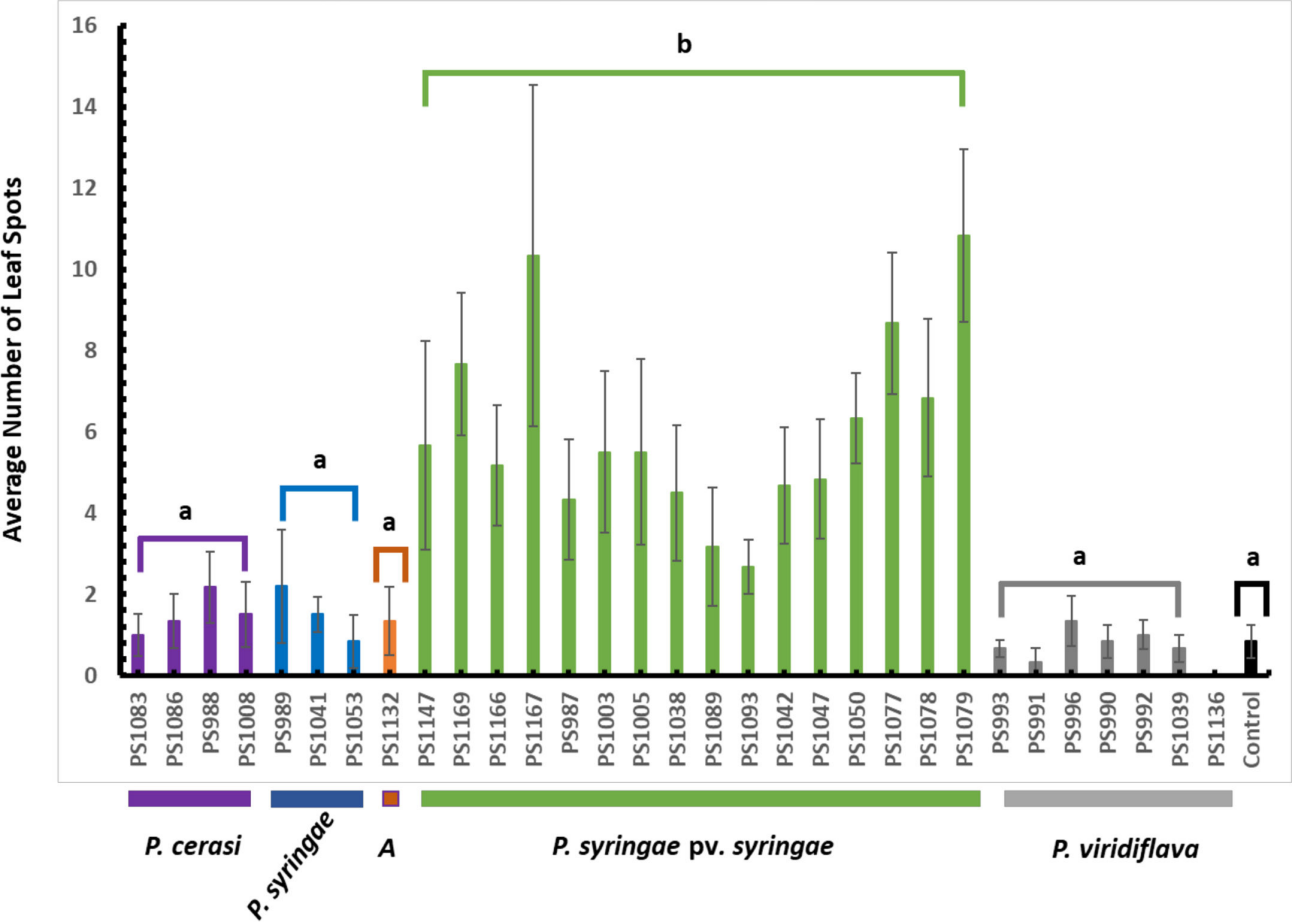
Results of leaf pathogenicity tests. Data are the average of all replications, and error bars indicate ±standard error of the mean. Different letters indicate significant differences (*P* < 0.01) in the disease ratings among isolates of the different genomospecies.

In pathogenicity tests using leaves in the field, isolates of the genomospecies *P. syringae* pv. *syringae* produced significantly (*P* < 0.01) more leaf spots in comparison to all the other genomospecies (Fig. 8). The leaf spots produced by isolates of genomospecies *P. syringae*, *P. cerasi*, *P. viridiflava,* and *A* were not statistically significant (*P* > 0.05) than the control (Fig. 8). Isolates of genomospecies *P. syringae* pv. *syringae* and *A* had genes encoding for *ahlI* and *ahlR* (Fig. 5). *ahlI* and *ahlR* are required to produce the quorum-sensing molecule AHL, which is crucial for epiphytic fitness (43, 44). Thus, isolates of these genomospecies are likely to survive, aggregate, and multiply on the leaf surface environment. For *P. syringae* sensu lato, the establishment of large epiphytic populations is a prerequisite for disease occurrence (41). Although both the isolates of genomospecies *P. syringae* pv. *syringae* and *A* had all the critical genes encoding for AHL (Fig. 5) suggesting they could potentially survive on the leaf surface longer, only *P. syringae* pv. *syringae* caused significant (*P* < 0.01) leaf spots (Fig. 8). This difference could be explained by the absence of genes encoding for phytotoxins syringomycin and syringopeptin in isolates of genomospecies *A* (Fig. 5). Syringomycin targets the plasma membrane of host cells inducing necrosis in plant tissue (51, 52). Like syringomycin, syringopeptin causes electrolyte leakage of plant cells resulting in the development of necrotic symptoms (53). Thus, isolates of genomospecies *A* likely survived and multiplied on the leaf surface, but they were not equipped with enough virulence determinants to induce any disease symptoms.

**Fig 9:**
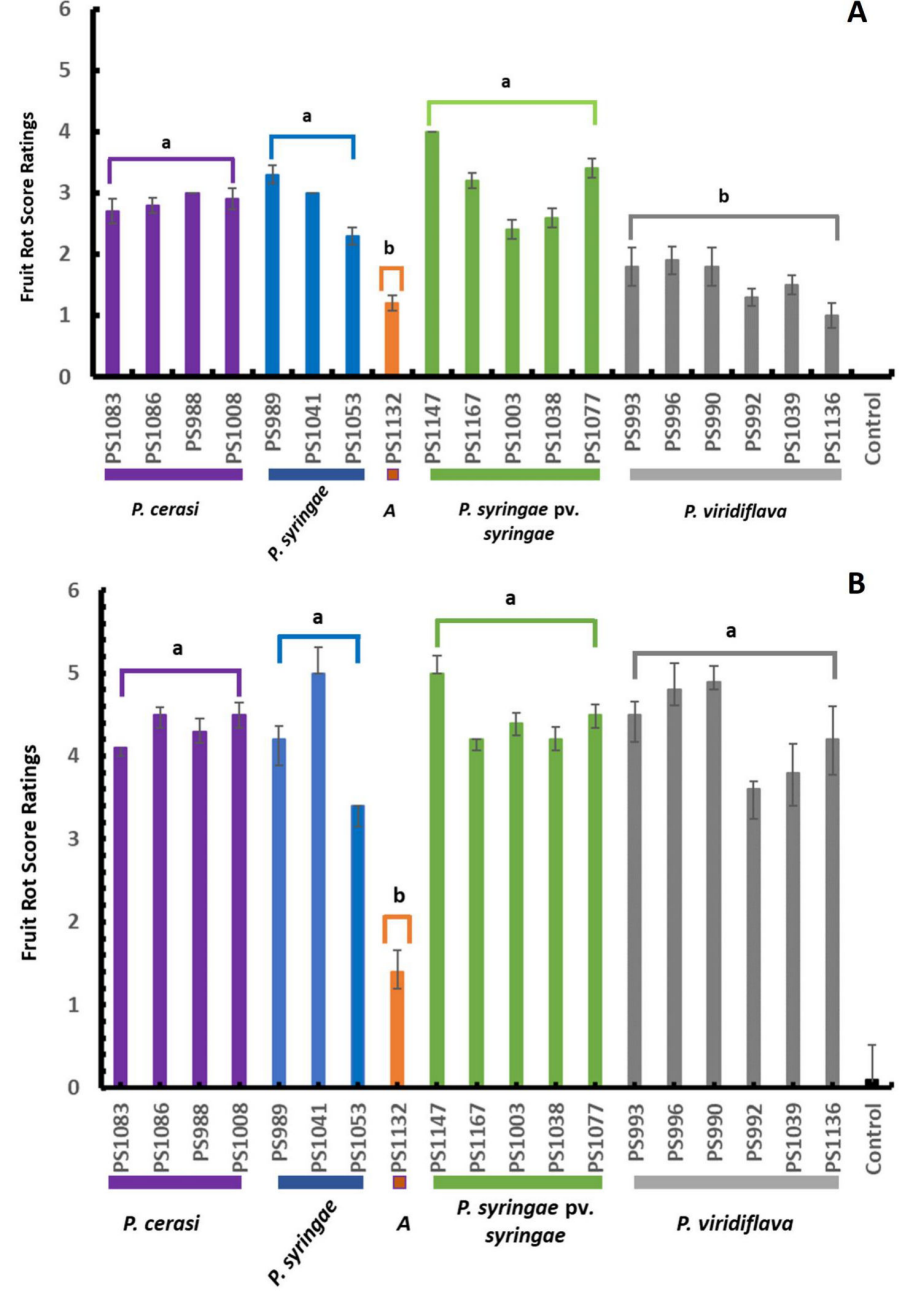
Detached immature fruit pathogenicity test. (**A**) Fruit rot score ratings 3 days post-inoculation. (**B**) Fruit rot score ratings 6 days post-inoculation. Data are the average of all replications, and error bars indicate ±standard deviation of the mean. Different letters indicate significant differences (*P* < 0.01) in the fruit rot score ratings among isolates of the different genomospecies.

In the detached immature, wounded cherry fruit pathogenicity assays, all isolates of the genomospecies *P. syringae* pv. *syringae*, *P. syringae*, *P. cerasi*, *P. viridiflava*, and *A* caused sunken black–dark brown lesions at the inoculated site (Fig. 9). There were variations in the symptom progression among isolates of the different genomospecies. Three days post-inoculation, isolates of genomospecies *P. syringae* pv. *syringae*, *P. syringae*, and *P. cerasi* had fruit lesions significantly (*P* < 0.05) larger than those of isolates from genomospecies *A* and *P. viridiflava* (Fig. 9A). Control treatments had no visible lesions on the fruits. At 6 days post-inoculation, there were no significant differences (*P* > 0.05) in the severity of symptoms among isolates of the genomospecies *P. syringae* pv. *syringae*, *P. syringae*, *P. cerasi,* and *P. viridiflava* (Fig. 9B). In contrast, symptoms caused by isolates from genomospecies *A* did not progress past the severity rating score of 2, and they were statistically less severe (*P* < 0.01) than symptoms produced by all the other genomospecies 6 days post-inoculation (Fig. 9B). Isolates of genomospecies *A* had no genes encoding for syringomycin and syringopeptin annotated in their genomic sequences (Fig. 5). Syringomycin and syringopeptin were shown to have a key role in disease development on fruits but were less important on leaves and wood (21). Isolates of genomospecies *A* naturally lacked genes encoding for phytotoxins syringomycin and syringopeptin, and the development of lesions on detached immature fruits was significantly reduced (Fig. 9). However, in contrast to the results of reference (21), our isolates did not cause disease on leaves and branches in the field (Fig. 6 to 8). This discrepancy could be explained by several factors. For example, we previously showed that laboratory leaf infiltration bioassays produce results distinct from the field assays, which was explained by the fact that laboratory assays are conducted in environments created to be highly conducive for disease development; however, such environments are rarely or never experienced in the field (5). Moreover, there were some variations in the methodology used including the cultivars used for testing. Regardless of variations in the methodology, the observed discrepancies emphasize the importance of field experiments over laboratory assays. To overcome the limitation associated with laboratory bioassays, we also inoculated immature cherry fruits attached to the plant without wounding. In this scenario, only isolates of *P. syringae* pv. *syringae* caused symptoms on fruits (Fig. S1E) with incidences of at least 80%. All the other genomospecies did not cause any disease symptoms. This could be attributed to *P. syringae* pv. *syringae* being a strong epiphyte. These contrasting results between field and laboratory experiments explain why mainly *P. syringae* pv. *syringae* has been reported as the causal agent of bacterial blast and bacterial canker of cherries in California. Although *P. cerasi* and *P. viridiflava* did not cause diseases in the field without wounding, they cannot be completely ignored as cherry pathogens. We observed several insects including the green stink bug (*Acrosternum hilare*) inciting wounds on immature cherry fruits. This can create entry points for weak epiphytes like *P. cerasi* and *P. viridiflava* if they are present at the time the insects incite the wounds. Moreover, these weak epiphytes can take advantage of unprotected pruning wounds and damage resulting from harvesting as entry points. More importantly, all isolates of *P. cerasi* and some isolates of *P. viridiflava* had genes encoding for INA (Fig. 5). Bacterial ice nucleation is associated with the ability to cause frost damage that may lead to water and nutrient release from plants and could create openings on the plant surface to facilitate bacterial entry (17).

### Genotype to phenotype correlations of antibiotic resistance

No isolates had genes or mutations that confer resistance to kasugamycin, an aminoglycoside antibiotic (Table 1). All isolates could not grow in the presence of 100 µg/mL kasugamycin, which is the label-recommended rate in cherry (Table 1). These results indicated that all isolates within the *P. syringae* species complex in our collection are susceptible to kasugamycin. A recent study of kasugamycin resistance in isolates from almonds in California also showed no resistance to kasugamycin (5) suggesting that currently in California resistance to kasugamycin is not a concern in stone fruit production. However, it is important to keep on monitoring for resistance as overreliance on antibiotics has been shown to result in the development of resistance. For example, prolonged use of kasugamycin to control bacterial brown stripe of rice caused by *Acidovorax avenae* subsp. *avenae* in Japan resulted in the emergence of resistant field isolates (54). Moreover, *in vitro* assays do not always correlate to the same effectiveness in the fields; thus, growers should properly follow all the manufacturer’s protocols to ensure optimal efficacy. For example, according to the label instructions, kasugamycin only works as a preventative measure; as a result, the timing of its application is critical for best results. Moreover, kasugamycin efficacy is reduced by photodegradation of the molecule (55), and growers need to consider this and avoid applications during times of the day with higher light intensity. We also tested for the susceptibility of isolates at a slightly lower rate of 75 µg/mL, and similarly, no isolates could grow (Table 1), an indication that kasugamycin can be successfully used for the management of bacterial blast and bacterial canker of cherry.

Genome mining also revealed that 16 of the 35 isolates (45.7%) belonging to the genomospecies *P. syringae* pv. *syringae* and 5 of the 11 isolates (45.5%) belonging to *P. viridiflava* had a *ctpV* gene (Table 1). CtpV is a putative copper exporter that reduces the impacts of copper toxicity and is involved in the virulence of *Mycobacterium tuberculosis* (48). *In vitro* copper sensitivity tests showed a positive correlation between the presence of the *ctpV* gene and copper resistance (Table 1). All isolates without the *ctpV* gene showed reduced growth in nutrient agar plates amended with 200 µg/mL MCE, and they could not grow at 300 µg/mL MCE (Table 1). In contrast, all isolates harboring the *ctpV* gene could grow even in nutrient agar amended with 400 µg/mL MCE (Table 1). This was consistent with our previous study in almonds, which surmised that the *ctpV* gene could be used to phenotype for copper resistance of *P. syringae* isolates (5). Our findings suggest copper might not be effective in the management of bacterial blast and bacterial canker of sweet cherry, and growers should consider other management strategies. In the future, it will be important to map out copper resistance in all cherry-producing counties of California. The use and overreliance on copper might have led to the development of resistance. Environmental-driven heterogeneity is common in bacteria (56); hence, there might be variation in resistance from one county to another or from one orchard to another. In the case of orchards where no copper resistance isolates are present, copper may be included as a rotational bactericide.

### Conclusions

*P. syringae* pv. *syringae* was the most frequently isolated genomospecies within the *P. syringae* species complex comprising 60% of the isolates classified as members of the *P. syringae* species complex. More importantly, it was the only genomospecies that caused symptoms in the field on both leaves and fruits. Isolates of the genomospecies *P. viridiflava*, *P. cerasi*, *P. syringae,* and *A* comprised 19%, 10%, 5%, and 3%, respectively, of the isolates classified as members of the *P. syringae* species complex. *P. viridiflava*, *P. cerasi*, *P. syringae*, and *A* did not cause any symptoms on the leaves or fruits in the field. The high frequency of isolation of *P. syringae* pv. *syringae* and the high virulence of most isolates of this genomospecies indicate it is the main causal agent of bacterial blast and bacterial canker of sweet cherry in California. Phylogenomic analyses did not identify any of the two *P. syringae* pv. *morsprunorum* races (now *P. amygdali* pv. *morsprunorum* and *P*. *avellanae*) previously reported from cherry, suggesting that these two species do not occur in California. It is possible, however, that we might have missed isolates of these genomospecies due to our isolation procedures. *P. cerasi*, *P. syringae*, and *P. viridiflava* caused cankers in the field just like *P. syringae* pv. *syringae* although *P. syringae* and *P. viridiflava* produced mild symptoms. This observation suggests these genomospecies are canker pathogens of sweet cherry and should be considered in diagnosis assays and management strategies of bacterial canker. More importantly, they have genes encoding for INA indicating their infection cycle might start through the blast phase of the disease. Isolates of the genomospecies *A* lacked genes encoding for phytotoxins syringomycin and syringopeptin and could not cause any disease symptoms. This suggests the phytotoxins syringomycin and syringopeptin play a crucial role just like the T3SS, and the synergistic effect of phytotoxins and T3SS is critical for symptom development at least in isolates used in this study. Our study emphasized the importance of field experiments over laboratory bioassays to better characterize the pathogenicity of *Pseudomonas* species. Although laboratory assays are relevant, they may overestimate the ability of organisms to cause disease due to optimized controlled conditions such as high humidity and optimal temperature. Based on results from this study, genomospecies *P. viridiflava* and *P. syringae* seem to be of less economic importance. Most importantly, we found no isolates resistant to kasugamycin suggesting this antibiotic can be successfully used for the management of bacterial blast and bacterial canker of cherry. On the other hand, there is a high frequency of isolates resistant to copper, and in some orchards, copper might not successfully be used for the management of bacterial blast and bacterial canker of sweet cherry. Field studies on control using copper may need to be conducted to understand if there is a loss of field control based on the high level of copper resistance observed in laboratory tests. Moreover, it will be helpful in future studies to further map out the frequency of copper-resistant isolates from all cherry-producing counties of California to better determine the benefit of copper in management strategies against bacterial blast and canker of sweet cherry.

## Data Availability

The raw sequencing data generated for this study were submitted to the National Centre for Biotechnology Information (NCBI) Sequence Read Archive under the BioProject ID: PRJNA1118275 with BioSample accessions SAMN41603515-SAMN41603593.

## SUPPLEMENTAL MATERIAL

Figure S1 legend spectrum.01324-24-s0001.docx Pathogenicity tests phenotypes.

Supplemental figure spectrum.01324-24-s0002.pdf Fig. S1.

Supplemental data spectrum.01324-24-s0003.xlsx Data S1-S5.

ASM does not own the copyrights to Supplemental Material that may be linked to, or accessed through, an article. The authors have granted ASM a non-exclusive, world-wide license to publish the Supplemental Material files. Please contact the corresponding author directly for reuse.
