# Supplementary material for: Pathogenicity, phylogenomic, and comparative genomic study of Pseudomonas syringae sensu lato affecting sweet cherry in California
Source: Microbiol Spectr. 2024 Sep 3;12(10):e01324-24. doi: 10.1128/spectrum.01324-24 (PMC11448091; doi:10.1128/spectrum.01324-24)
Supplement: Figure S1 legend — Pathogenicity tests phenotypes. [file spectrum.01324-24-s0001.docx]

**Supplementary Figure 1. Pathogenicity tests phenotypes** (A) a branch showing 3 punched points and no gumming (B) a branch showing 3 punched points with little gumming, this was typical for isolates of genomospecies *P. viridiflava* and *P. syringae* (C) a branch showing 3 punched points with clear and larger amber colored gumballs, this was typically produced by isolates of genomospecies *P. syringae* pv. *syringae* and *P. cerasi* (D) an image showing leaf spot symptoms (E) fruit rot lesions produced by isolates of *P. syringae* pv. *syringae* on detached unwounded immature cherry fruits.
