# Supplementary figures and images for: Pathogenicity, phylogenomic, and comparative genomic study of Pseudomonas syringae sensu lato affecting sweet cherry in California
Source: Microbiol Spectr. 2024 Sep 3;12(10):e01324-24. doi: 10.1128/spectrum.01324-24 (PMC11448091; doi:10.1128/spectrum.01324-24)

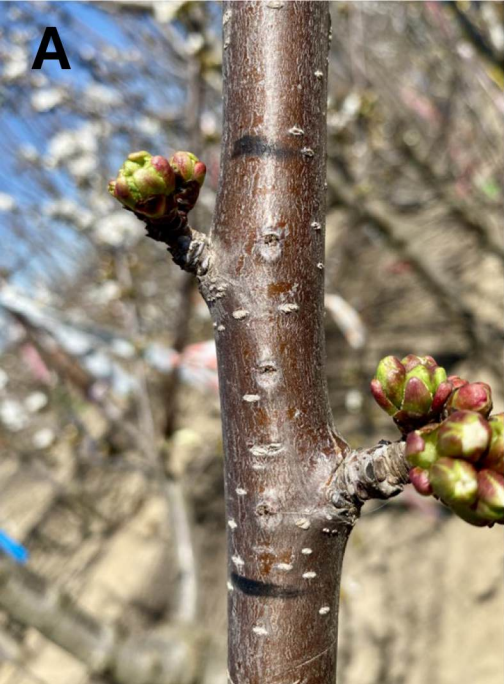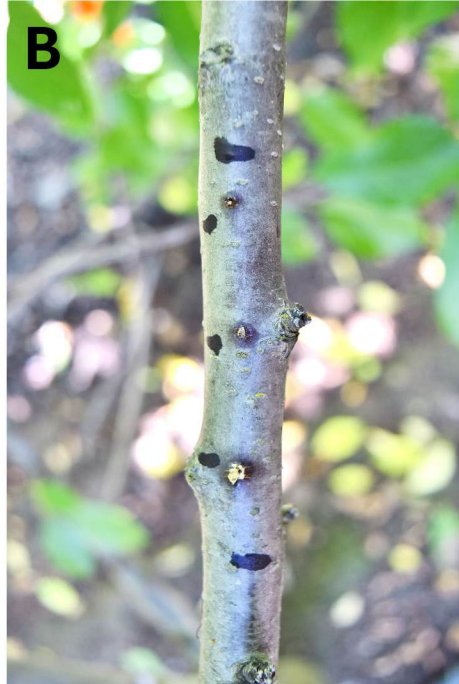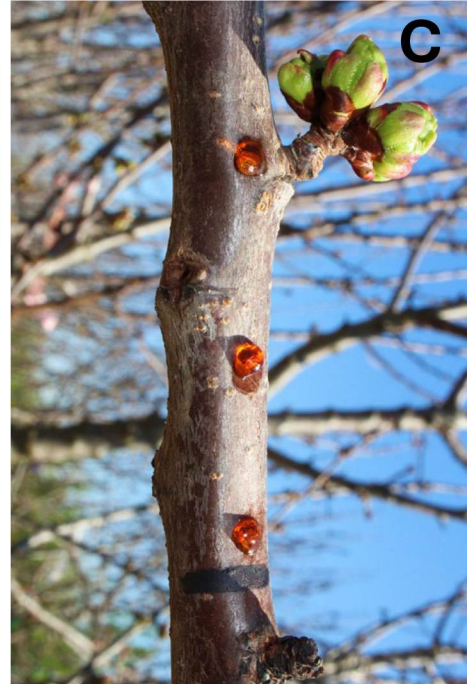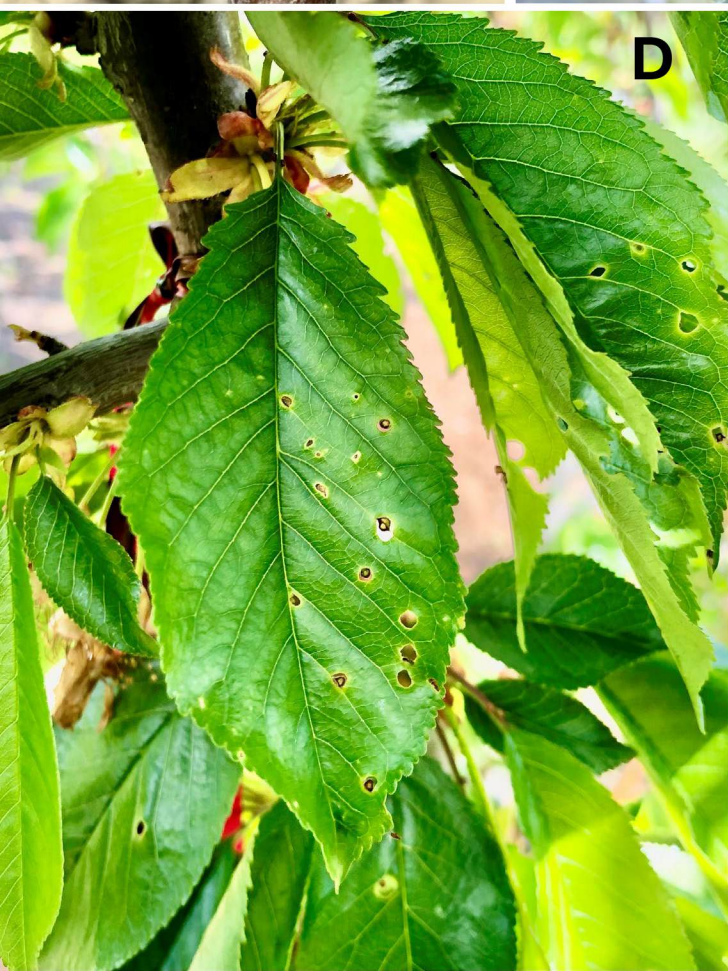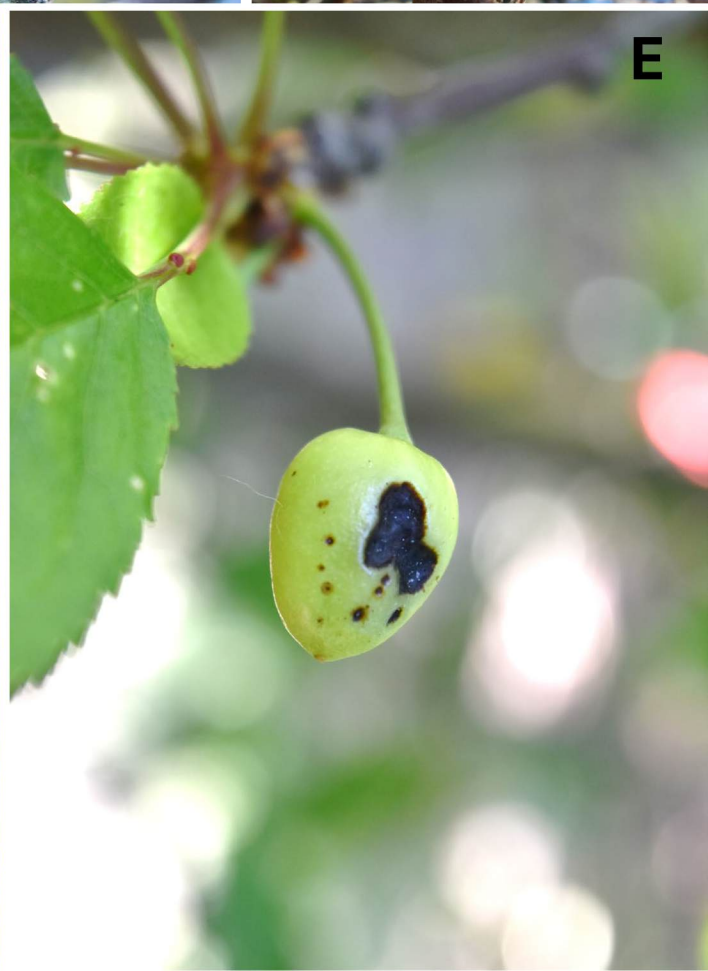

Supplement: Supplemental figure — Fig. S1. [file spectrum.01324-24-s0002.pdf]
